# A Comparative Study on the Role of Polyvinylpyrrolidone Molecular Weight on the Functionalization of Various Carbon Nanotubes and Their Composites

**DOI:** 10.3390/polym13152447

**Published:** 2021-07-25

**Authors:** Muthuraman Namasivayam, Mats R. Andersson, Joseph G. Shapter

**Affiliations:** 1 School of Engineering, Information Technology and Physical Sciences, Gippsland Campus, Federation University, Churchill, VIC 3842, Australia; m.namasivayam@federation.edu.au; 2 College of Science and Engineering, Flinders University, Bedford Park, Adelaide, SA 5042, Australia; mats.andersson@flinders.edu.au; 3 Australian Institute for Bioengineering and Nanotechnology, The University of Queensland, St. Lucia, Brisbane, QLD 4072, Australia

**Keywords:** polyvinylpyrrolidone, polymer nanocomposites, non-covalent functionalization, carbon nanotubes, physical properties

## Abstract

Polyvinylidene fluoride (PVDF) nanocomposites filled with polyvinylpyrrolidone (PVP) wrapped carbon nanotubes were prepared via a solution casting technique. The effect of the molecular weight (polymer chain length) of the PVP on the ability to wrap different nanotube structures and its impact towards nanotube dispersibility in the polymer matrix was explored. The study was conducted with PVP of four different molecular weights and nanotubes of three different structures. The composites that exhibit an effective nanotube dispersion lead to a nanotube network that facilitates improved thermal, electrical, and mechanical properties. It was observed that nanotubes of different structures exhibit stable dispersions in the polymer matrix though PVP functionalization of different molecular weights, but the key is achieving an effective nanotube dispersion at low PVP concentrations. This is observed in MWNT and AP-SWNT based composites with PVP of low molecular weight, leading to a thermal conductivity enhancement of 147% and 53%, respectively, while for P3-SWNT based composites, PVP of high molecular weight yields an enhancement of 25% in thermal conductivity compared to the non-functionalized CNT-PVDF composite.

## 1. Introduction

The field of polymer nanocomposites had been of interest for the past few decades, and there is an increasing demand for more advanced functional materials. Although carbon nanotubes act as an effective filler, aggregate formation in the polymer matrix due to the high van der Waals interactions between the nanotubes is inevitable. Surface functionalization of nanotubes is the best approach to reduce the interaction between nanotubes while preserving the original morphology and conductivity of the nanotubes. This can also provide stable dispersions in organic solvents. Non-covalent functionalization (polymer wrapping) of nanotubes is considered a highly effective way to overcome aggregate formation and achieve a homogeneous dispersion of nanotubes in the polymer matrix without disrupting the intrinsic characteristics of the nanotubes. The physical and mechanical properties of polymer nanocomposites can be influenced by both the molecular weight of the polymer and the structure of the nanotube filler.

Recent work on the development of polymer nanocomposites has seen a strong emphasis on the use of organic, inorganic, or carbon fillers [1], leading to materials with valuable engineering applications [2]. Nanoscale fillers, such as carbon nanotubes, graphene, and nanocellulose allow control and enhancement of the physical properties of polymer nanocomposites [3]. The high modulus, high electrical and thermal conductivity, and large surface area of carbon nanotubes has drawn much attention since their discovery in 1991 [4,5], and significant efforts towards improvement of strength and conductivity of polymers through incorporation of nanotubes has been reported [6,7]. Thermal conductivity measurements predict a value between 200 W·m^−1^·K^−1^ and 3000 W·m^−1^·K^−1^ for multi-walled nanotubes, and above 2000 W·m^−1^·K^−1^ for single-walled nanotubes. Carbon nanotubes possess a Young’s modulus of ~1 TPa, a tensile strength of tens of GPa, and a fracture strain of ~5 to 12% [8]. The development of CNT based polymer composites has shown remarkable property enhancement at low filler concentration compared to conventional composites [9].

Superior piezoelectric and pyroelectric properties of semi-crystalline polyvinylidene fluoride (PVDF) has opened a range of commercial and technological applications, including supercapacitors, transducers, and actuators for batteries [10]. Nanotube filled PVDF composites have drawn much attention due to their low cost and processability, and much work towards understanding the effect of nanotubes in PVDF composites have been reported [11,12]. Application for these conducting polymers in the field of electronics are extensive, ranging from flexible sensors and nanogenerators to hydrophobic membranes and ultrafiltration membranes for wastewater management [13], and tends to improve fouling resistance in PVDF membranes during membrane filtration processes [14].

The performance of nanotube filled polymer composites depends mainly on the dispersion of nanotubes in the polymer matrix, and the major issue with nanoscale fillers is their inability to disperse effectively in a polymer matrix. Strong van der Waals interactions lead to clustering or agglomeration of nanotubes in the matrix, and the extent of cluster formation depends on the amount of filler or filler geometry [15]. The tendency for agglomeration grows with filler loading and/or the aspect ratio of the filler. Although different mechanical and chemical techniques to overcome agglomeration have been developed over the years, some degree of agglomeration persists in highly loaded composite materials [16]. Martins et al. investigated the morphology, rheological behavior, and electrical conductivity of multi-walled nanotube (MWNT) filled PVDF composites. It was observed that a lower content (0.5 wt. %) of MWNTs presented a uniform dispersion through the PVDF matrix, whereas 1 wt. % of MWNTs is observed to present a percolated network. For the nanocomposites with 2 and 5 wt. % loading, the formation of network structure was clearly evident. No significant change in electrical conductivity is observed when nanotube content is above 2 wt. %. However, at 5 wt. % loading, a significant change in rheological behavior can be observed, which can make the processing of these nanotubes difficult [17]. This might be attributed to the fact that the processing of nanotubes at higher concentration might be necessary in order to achieve an effective nanotube structure. Similarly, Ke et al. conducted a study investigating the electrical conductivity of a carbon nanotube (CNT) and carbon black (CB) filled PVDF composite. The result showed that an increasing content of carbon black leads to an improvement in electrical conductivity, even in composites with content of carbon nanotubes below the percolation threshold. This is attributed to the fact that the CB particles bridge the gap between nanotubes, forming a hybrid conductive network when the CNT content is not high enough to form conductive paths themselves. However, in composites with higher CNT content, the addition of CB particles only makes the already existing conductive network more complex [18]. Moreover, a higher amount of CB particles might compromise the transparency of the composite film. In general, conductive networks are sparse in composites with nanotubes nearer to percolation threshold or with agglomerated nanotubes. Therefore, the presence of a nanotube network structure alone would not yield an effective conductive polymer nanocomposite, but the compactness of filler networks and connection among nanotubes plays a crucial role. Ma et al. reported that, although mechanical dispersion methods such as ultrasonication, ball milling, and calendering processes may have proven to be effective, they can damage the CNT structure, and these tools may not be suitable for dispersion in all types of polymer matrices [19]. However, functionalization of nanotubes proved to be an effective way to improve the thermal performance of the composite by forming a soft interface between the nanotubes and the polymer matrix, thus decreasing the thermal resistance at the interface. There are various approaches for CNT functionalization, including defect functionalization, covalent functionalization, and non-covalent functionalization [20].

Defect functionalization is a method that creates defect sites on side walls and the open ends of CNTs as a result of strong acid treatment. However, the defect sites created by this method are extremely sparse. Additionally, the CNTs are broken into shorter tubes in this oxidisation method, which not only compromises the aspect ratio but also the overall thermal conductivity [21]. In case of covalent functionalization, the translational symmetry of CNT sidewall is disrupted with a change of hybridization from sp^2^ to sp^3^ carbon atoms and a simultaneous loss of π conjugation system of the graphene layer [22]. However, a major negative impact towards covalent functionalization is that the CNTs are prone to formation of defect sites along the sidewalls and, in extreme cases, the structure of the nanotube is highly disrupted [23]. Furthermore, the use of concentrated acids is not environmental friendly [24]. However, non-covalent functionalization of nanotubes has proven to be an effective way to improve the interaction between nanotubes and a polymer matrix, forming a network structure without causing damage to the structure of the nanotubes [25].

A strong π-π interaction allows adsorption of conjugated polymers on the CNT surfaces in the form of wrapping, thus creating a supramolecular complex. The chains of PVP wrap themselves around the surface of nanotubes due to strong hydrophobic interaction between the CNT surface and the PVP hydrophobic tail, exposing the hydrophilic lactam group to the solvent. The presence of PVP on the CNT surface provides a steric hindrance effect that weakens the van der Waals interactions between nanotubes and increases dispersibility of CNTs in various polymers [26]. Other forms of polymer wrapping interaction including CH-π and Cation-π that, although comparatively weaker, are capable of forming stable CNT-polymer dispersions. The aqueous solubility of CNTs can be improved through polymer wrapping in water soluble polymers such as polyvinylpyrrolidone (PVP). Polyvinylpyrrolidone is a non-toxic, non-ionic polymer with C=O, C–N, and CH_2_ functional groups that contains hydrophilic side groups (pyrrolidone moiety) and a hydrophobic backbone, making PVP soluble in both aqueous and non-aqueous solvents. A hydrophobic group (the alkyl group), with repulsive forces that arise from its hydrophobic carbon chain, extends into the solvent and interacts with each other, preventing aggregation of nanoparticles and nanotubes. Moreover, PVP also acts as a hydrophilic modifier to mitigate membrane fouling [27] and is a great stabilizer [28]; furthermore, PVP addition tend to have a dramatic change towards the structure of PVDF [29]. This is observed in a study on high permeability graphene oxide and polyvinylpyrrolidone blended PVDF membranes; PVP acts as a good pore former, increases membrane porosity and thus water flux. However, an optimum benefit is only achieved in a suitable PVP content of 3–4 wt. % of the casting solution and, at higher ratio PVP, blended membranes tend to encounter denser structure formation [30]. Zhang et al. studied the thermal conductivity of PVP treated MWNTs in PVDF composite and confirmed that the presence of PVP greatly improves the dispersion of CNTs in the PVDF composite. The result obtained showed that a denser CNT network structure is observed in PVP treated MWNT/PVDF composite compared to untreated MWNT/PVDF composite of the same nanotube loading [31]. However, a recent report on Ag nanoparticles capped with PVP oligomers revealed that the length of the chain played an important role in stabilizing the nanoparticles. A longer chain is expected to provide enhanced stability, but the increase in the length of the PVP chain primarily leads to the formation of an additional external layer around the nanoparticle, leading to a higher surface coverage [32]. Similarly, in a recent study by Zhang et al. on the preparation of PVP assisted highly conductive and durable nanofiber composite, it was revealed that the PVP layer could not only enhance the interfacial interaction between the Ag nanoparticles (AgNPs), leading to enhanced mechanical properties, but also forms a protective layer around AgNPs that prevents oxidizing [33]. A study by Uthaman et al. on the mechanical and water uptake properties of PVP modified MWNTs in epoxy matrix revealed that an obvious increase in the mechanical and thermal properties is observed with PVP surface modification. However, the weight concentration of PVP-CNTs in epoxy plays a crucial role, and it is recommended that an optimal concentration of 1.5% must not be exceeded to ensure enhanced mechanical properties, while at higher weight concentration of 2%, CNTs tend to agglomerate [34]. In a similar study, an enhancement in thermal conductivity is achieved by adding carbon nanotubes in a Polyethylene glycol (PEG) and polyvinylpyrrolidone (PVP) composite of equal mass ratio. Increasing the concentration of nanotube from 1 wt. % to 5 wt. % by maintaining a constant PEG and PVP concentration enhances the thermal conductivity, but a further increase leads to CNT agglomeration. The concentration of PVP did not seem to have an impact on the CNTs beyond a certain concentration and, eventually, the relation between the concentration of PVP and CNTs tend to have an impact on the thermal outcome of the overall composite [35].

Most of these reports use PVP to try to effectively distribute the carbon nanotubes in the polymer composite. Hence, an understanding of the interplay between PVP molecular weight and the structure of the nanotubes is critical to ensure the CNTs are dispersed in the composites. In this work, PVDF nanocomposites filled with PVP wrapped carbon nanotubes were prepared via a solution casting technique. The research focuses on investigating the influence of CNT morphology towards polymer wrapping and its effect on the physical properties of the overall nanocomposites. The study is conducted with PVP polymers of increasing molecular weight and nanotubes of three different morphologies, namely, As-Prepared Single-Walled Nanotubes, P3-Single-Walled Nanotubes, and Multi-Walled Nanotubes. The research concentrates on the change in polymer wrapping behavior with difference in nanotube morphology and polymer chain length. Unlike previous reports where the loading of CNTs has typically been changed, in this work the CNT loading will be held constant while the loading of the wrapping polymer is varied.

## 2. Materials and Methods

The multi-walled carbon nanotubes used in this research were purchased from Sigma–Aldrich (Product No.: 755133, St. Louis, MO, USA) with an average diameter of 9.5 nm, a length of 1.5 μm, and an impurity of less than 5% metal oxide. AP-SWNTs (As prepared) and P3-SWNTs were purchased from Carbon Solutions (Riverside, CA, USA), with an individual tube length ranging from 0.5–3 μm and an average diameter of 1.4 nm. The AP-SWNTs used in this research are about 60–70% pure with a metal content of less than 30%, a bundle length of 1 to 5 μm, and a bundle diameter range of 2 to 10 nm. The P3-SWNT used were more than 90% pure with the presence of 5–7% metal content and a bundle length of 500 nm to 1.5 μm (~1 μm) with a diameter range of 4–5 nm. The commercial P3-SWNTs and MWNTs were purified through acid treatment with nitric acid.

PVDF with a melt flow rate (MFR) of 20–35 g per 10 min (230 °C, 3.8 Kg^−1^), density of 1.78 g·mL^−1^ at 25 °C, and an average molecular weight of 180,000 g·mol^−1^ (Sigma–Aldrich, St. Louis, MO, USA) was used as the polymer matrix. Polyvinylpyrrolidone with molecular weights of 10,000 g·mol^−1^, 40,000 g·mol^−1^, 55,000 g·mol^−1^, and 360,000 g·mol^−1^ (Sigma–Aldrich) were used for the non-covalent functionalization of nanotubes.

### 2.1. Sample Preperation

Prior to use, all the nanotubes underwent a dilute nitric acid (3M HNO3) reflux for 16 h to achieve a high level of purity. The resultant carbon nanotubes were filtered and dried overnight in an oven with a temperature ranging from 80 °C to 90 °C. This nitric acid treatment is a well-established purification approach for nanotubes. It removes any remaining metal catalyst particles and small non-nanotube carbon fragments. It does not damage the nanotubes nor change the level of oxidation of the sample. Concentrated acids are required to cut the nanotubes and increase the levels of oxidation. The P3-SWNTs will have the highest level of oxidation followed by the MWNTs and then the AP-SWNTs, which we would expect to have a low oxygen content.

The nanocomposites were prepared through a solution mixing method, which includes two steps.

The first step was to obtain the “solution” containing polymer wrapped CNTs and the second step was to mix the “solution” and the PVDF polymer in the same solvent, followed by a controlled evaporation process through deposition over a glass or silicon substrate, resulting in a film thickness of approximately 40 μm. An Elmasonic S30H 280W (Techspan Australia, Kenmore, Queensland) power bath sonicator was used for the sample preparation.

First step: 1 mg of unmodified CNTs was suspended in 250 μL of DMF and sonicated for 10 min to produce a uniform dispersion [36]. Then, a defined amount of functionalization polymer (PVP) was dispersed in the CNT solution. The mixture was sonicated for a period of 45 min and then left undisturbed overnight in order to ensure that the prepared solution sustained the dispersion without re-aggregation for a period of 12–15 h [37,38].

Second step: The mixture was combined with 20 mg of PVDF polymer in 200 μL of DMF and sonicated for a period of 4 h [39]. The resulting non-covalently functionalized PVP@CNT/PVDF mixture was then deposited on a clean silicon/glass wafer of about 2 cm in length and 2 cm in breadth and dried in an oven at a temperature of 100 °C for 24 h.

In this work, the amount of CNT and PVDF remained essentially constant, with only the concentration of functionalization polymer varying from a weight percent of 1.48% to 41.18% (0.025 mg to 0.7 mg) with respect to the amount of CNT used to make the composite. Every sample was prepared independently thrice, and the average result was plotted in the graphs, with error bars obtained from the standard deviation. A comparative result was produced.

### 2.2. Characterization

#### 2.2.1. Thermal Conductivity

Thermal conductivity was measured using a steady state technique in a well-insulated chamber, where the sample was placed between a heat source and a heat sink with a known amount of heat supplied through a steady state power input using a PID Temperature controller (Ocean Controls N322, Carrum Downs, Australia). The temperature difference across a given length of the sample was measured using a differential temperature meter (Fluke 52 II) after a steady-state temperature distribution was acquired. Thermal conductivity of the sample was calculated using Fourier’s Law of Heat Conduction:

(1) $$k=\frac{\mathrm{qL}}{A\Delta T}\;(W\cdot m^{-1}\cdot K^{-1})$$

where q is the amount of heat supplied through the sample and A is the cross-sectional area of the sample. L is the distance through which heat flows and ΔT is the temperature difference observed.

#### 2.2.2. Electrical Conductivity

Nanocomposites were deposited on a clean glass substrate and electrical conductivity was measured at room temperature using a four-point probe method.

Sheet resistivity was measured using the voltage and current reading from the probe:

(2) $$Sheet\;resistivity\;\left(\frac{\rho }{□}\right)=\left(\frac{\Omega }{sq}\right)=\frac{\pi }{\ln\left(2\right)}\cdot \frac{V}{I}$$

where:

$$\frac{\pi }{\ln\left(2\right)}=4.532$$

In typical usage, the current was set to 4.53 mA so that the resistivity is simply the voltage reading in mV.

Electrical conductivity of the composite was measured to be:

(3) $$S/m=\frac{1}{\left(R*t\right)}$$

R is in ohms/sq. and t is film thickness in m.

#### 2.2.3. Differential Scanning Calorimetry

A TA Instruments 2930 DSC (New Castle, DE, USA) was used to investigate the crystallization and melting behavior of the sample. A sample of about 9 mg was first heated from a temperature of 20 °C to 200 °C and then maintained at 200 °C for one minute before cooling down from 200 °C to 20 °C at a rate of 10 °C min^−1^. The steps were repeated to acquire a second heating scan. The degree of crystallinity was obtained from the enthalpy of melting Δ*H_f_*, the enthalpy of crystallization Δ*H_c_*, and the enthalpy of melting for a fully crystalline polymer Δ*H_f_*_,100%_.

(4) $$\mathrm{Degree}\;\mathrm{of}\;\mathrm{crystallinity}\;=\;\frac{\Delta H_{f}\;-\Delta H_{c}}{\Delta H_{f},100\%}\;*\;100$$

#### 2.2.4. Dynamic Mechanical Analyser

The polymer nanocomposite samples prepared had a thickness of about 40 microns, and a TA Instruments Q800 DMA (New Castle, DE, USA) was used to analyze the mechanical properties of the sample. The mode of oscillation used was Tensile Strain and the procedure performed was temperature ramp with a frequency sweep. The experiments were performed in a temperature range of −90 °C to 200 °C at a temperature ramp of 3 °C·min^−1^ with a soak time of 1 min, meaning the sample is maintained at −90 °C for 1 minute to make sure the sample actually reached the temperature before the experiment began. An amplitude of 20 μm and a static force of 0.05 Newton were applied and maintained throughout the experiment. Storage modulus and Tan delta of the composite were characterized as a function of temperature and compared. Selected samples were measured to probe the changes between samples that showed thermal conductivity improvements and those that did not. Where samples displayed very similar thermal conductivity behavior (for example, MWNTs with PVP_10000_ above 15 wt. %), just one or two representative samples were measured. The samples with the highest thermal conductivity for each set were always plotted in red.

## 3. Results and Discussion

Nanotubes of three different structures were non-covalently functionalized using polyvinylpyrrolidone polymers of different molecular weight towards the preparation of PVP@CNT/PVDF composites at a range of PVP concentrations, and the thermal conductivity of each sample was measured. The measurement observed at 0 wt. % is the thermal conductivity of non-functionalized MWNT or P3-SWNT or AP-SWNT/PVDF composite. The theoretical thermal conductivity value of a pure PVDF polymer is 0.2 W·m^−1^·K^−1^ [40,41] and the presence of nanotubes in the PVDF matrix, irrespective of the nanotube structure, enhances the thermal conductivity of the polymer composites. However, the structure of the nanotube influences the degree of thermal conductivity enhancement. AP-SWNT/PVDF composites with 0 wt. % PVP exhibited the highest thermal conductivity observed. With a value of about 4.37 W·m^−1^·K^−1^, a lower thermal conductivity with a value of 2.93 W·m^−1^·K^−1^ was observed with unfunctionalized P3-SWNT/PVDF composite, and MWNT/PVDF composites exhibited the lowest thermal conductivity value of about 1.48 W·m^−1^·K^−1^ for the composites. The high value observed with AP-SWNTs can be attributed to their high aspect ratio, considering the fact that the thermal and mechanical properties of a nanocomposite are highly influenced by the filler dimensions. Moreover, strong interfacial bonding between the polymer matrix and CNT is highly influenced by the aspect ratio of the CNTs. Although a similar diameter was observed for AP-SWNT and P3-SWNT, the average length of the AP-SWNT is twice than that of P3-SWNT. This could be due to the fact that as purchased P3-SWNTs are a highly oxidized and purified form of AP-SWNTs and, as a result, the presence of carboxyl content in a P3-SWNT is higher than in AP-SWNT. This leads to formation of defect sites and a decrease in the length of P3-SWNT, which in turn leads to a reduction in thermal conductivity. MWNTs are also achieved after a series of acid treatments in order to attain a highly purified and oxidized form, thus causing defect sites that affects thermal conductivity. Additionally, M. Fujii et al. reported that the interaction of phonons and electrons between the multi-walled layers of a nanotube affects the thermal conductivity [42], meaning the higher number of nanotube layers in MWNTs allow for increased phonon scattering, leading to a decrease in thermal conductivity compared to SWNTs.

With these baseline data measured, the various CNTs were now first non-covalently functionalized by wrapping them with PVP of various molecular weights before they were used to form the composites. The data in the following sections shows that the thermal conductivity of the composites can be enhanced significantly, but only for certain combinations of CNT structure and PVP molecular weight. The combinations that yield enhanced properties show significant improvements in mechanical strength, demonstrating the more effective dispersion of the CNTs in the composites. Additionally, in all cases, a high loading of PVP lowers the electrical conductivity. The increasing layer thickness of the PVP at higher loadings leads to isolation of the CNTs, each one preventing establishment of a percolation network, thus lowering the electrical conductivity.

### 3.1. PVP_10000_ Functionalized CNT in PVDF Composite

**Figure 1:**
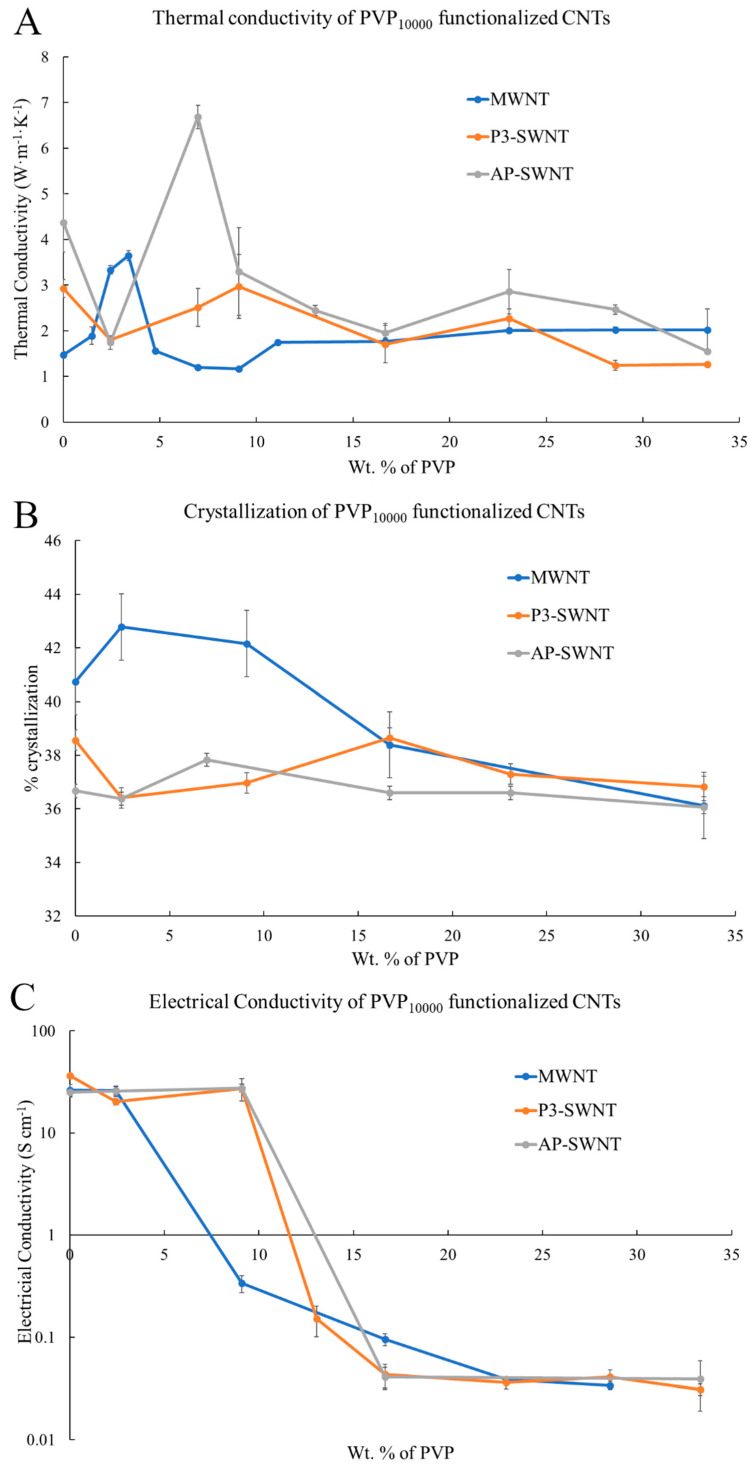
(**A**) Thermal conductivity of three different types of PVP_10000_ functionalized CNT/PVDF composites; (**B**) Percent Crystallization; and (**C**) Electrical conductivity of PVP_10000_ functionalized CNT/PVDF composites at different PVP concentrations.

PVP_10000_ functionalization exhibit an effective enhancement in thermal conductivity with both AP-SWNTs and MWNT based PVDF composite at low concentrations of PVP (see Figure 1A). An increase in thermal conductivity from a value of about 1.48 W·m^−1^·K^−1^ to a value of 3.65 W·m^−1^·K^−1^ at a PVP concentration of 3.38 wt. % was observed for the PVP_10000_ functionalized MWNT/PVDF composite, which is a 146% increase. PVP_10000_ functionalization also exhibited an increase in thermal conductivity, with the AP-SWNT/PVDF composite at a concentration of 6.98 wt. % exhibiting a 52.86% enhancement from a value of about 4.37 W·m^−1^·K^−1^ to a value of 6.68 W·m^−1^·K^−1^. However, such an enhancement was not observed with P3-SWNTs in the low concentration range. This shows the inability of the shorter polymer chain to achieve a stable dispersion of P3-SWNTs in the polymer matrix. In polymer functionalization, the polymer wraps around the surface of the nanotubes, forming a supramolecular complex thus enhancing the dispersibility of nanotubes in organic solvents, and the polymer chain length of PVP_10000_ is not compatible to cover enough surface area of P3 single-walled nanotubes to achieve dispersion at low PVP concentration. This can be attributed to the difference in the aspect ratio of AP and P3 SWNTs. The lower aspect ratio of P3-SWNT leads to a higher number of individual P3 single-walled nanotube present in the polymer matrix compared to AP single-walled nanotubes of the same mass. Thus, for P3 SWNT based composites, the PVP polymer has to functionalize a higher number of nanotubes to produce an effective dispersion, which was not achieved with a short polymer chain length of PVP_10000_ at low concentrations. However, at higher concentrations, PVP_10000_ may enable an efficient surface coverage, leading to a higher dispersion in the polymer matrix.

For both AP-SWNTs and MWNT, at loadings of PVP_10000_ above those that led to enhanced thermal conductivity, the conductivity returns to values very similar to those observed for non-functionalized nanotubes. This was expected as the polymer wrapping is a balancing act between having enough wrapping to improve dispersion in the PVDF while still allowing a network of nanotubes to form and having too much wrapping, which might still help the extent of dispersion in the PDVF but isolate the nanotube bundles from each other, thus limiting the effectiveness of their inclusion.

The crystallinity (Figure 1B) and electrical conductivity (Figure 1C) were measured and provide insights into the thermal conductivity results observed in Figure 1A. PVP_10000_ functionalization leads to a 5% and 3.16% increase in the degree of crystallization with MWNT and AP-SWNT based polymer composites. However, PVP_10000_ functionalization does not tend to exhibit any effective dispersion in P3-SWNT based composites at PVP concentrations lower than 33.33 wt. % that would trigger a nucleation effect for the entire polymer matrix, but the possibility of an improvement in dispersion at higher PVP_10000_ concentrations cannot be discounted.

The electrical conductivity (Figure 1C) result for PVP_10000_ functionalized AP-SWNT based composites showed an increase in value from 25.1 S·cm^−1^ to about 27.6 S·cm^−1^, along the same concentration range that exhibited a thermal conductivity enhancement. Similarly, the electrical conductivity observed with PVP_10000_ functionalization in MWNT based composite exhibited a stable conductivity value along the low concentration range that displayed a thermal conductivity increase, confirming the presence of a conductive nanotube network. However, the formation of such a conductive network was not observed with the PVP_10000_ functionalized P3-SWNT based composites, leading to a lower electrical conductivity value compared to the unmodified nanotube composite at low PVP concentrations, and it continue to decrease even further with increasing concentrations.

**Figure 2:**
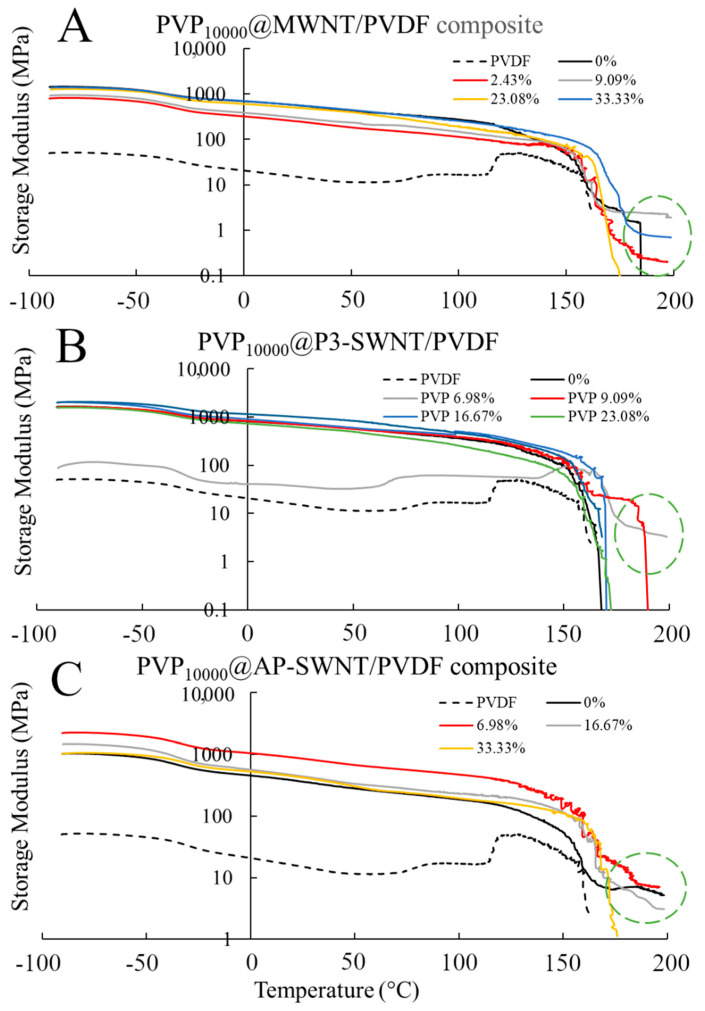
Storage Modulus of PVP_10000_ functionalized MWNT (**A**); P3-SWNT (**B**); and AP-SWNT (**C**) in PVDF composite.

The mechanical strength of composites is highly dependent on the presence of a nanotube network, and an effective dispersion of nanotubes would enhance the interaction between the nanotubes and PVDF polymer, leading to an increase in the storage modulus and subsequently its mechanical strength. An effective dispersion of nanotubes was observed with PVP_10000_ functionalization of the MWNT based PVDF composite at a concentration range of 2.44 wt. %, leading to a fairly similar storage modulus as an unmodified nanotube composite, as observed in Figure 2A, yet the functionalized composite tends to exhibit strong mechanical strength, even at a comparatively higher temperature of about 197 °C. This could be attributed to an enhanced interaction of nanotube and polymer matrix, confirming the presence of a stable nanotube network even at higher temperatures. Similarly, the PVP_10000_ functionalized AP-SWNT nanotube composite exhibits a high storage modulus at a low concentration range, and the highest storage modulus compared to unmodified nanotube composite is observed for the composite functionalized at a PVP concentration of 6.98 wt. % (Figure 2C), which correlates with the thermal conductivity result observed in Figure 1A. In line with other results, the storage modulus of the PVP_10000_ functionalized P3-SWNT composite (Figure 2B) correlates with the thermal conductivity observed for this composite. For P3-SWNT, the thermal conductivity result showed small increases in composites functionalized with PVP concentrations of 9.09 wt. % and 23.1 wt. %, but they were not significantly enhanced compared to the thermal conductivity of an unmodified P3-SWNT composite, as observed with MWNT or AP-SWNT based composites. These P3-SWNT composites also tend to exhibit an enhancement in their mechanical strength, as highlighted in the circle in Figure 2B. This could be explained with the hypothesis that the PVP_10000_ functionalization dispersed a small amount of nanotubes in the composite, causing a mild improvement in their dispersion and nanotube network formation, leading to small hike in their thermal conductivity and mechanical strength. However, a stable dispersion of nanotubes in the overall composite is yet to be achieved because of the lack of sufficient nanotube functionalization at low PVP concentration due to the short length of the PVP_10000_ polymer. The fact that this was only observed with P3-SWNTs based composites in Figure 1A and Figure 2B could be due to the short length of P3-SWNTs compared to MWNTs and AP-SWNTs. It could also be due to the fact that, unlike MWNT and AP-SWNT based composites, an effective dispersion of the overall composite is only expected at higher PVP_10000_ concentrations for P3-SWNT based composites, which might lead to enhancements in thermal conductivity or crystallization similar to those observed for MWNT and AP-SWNT based composites at low PVP_10000_ concentrations.

### 3.2. PVP_40000_ Functionalized CNT in PVDF Composite

**Figure 3:**
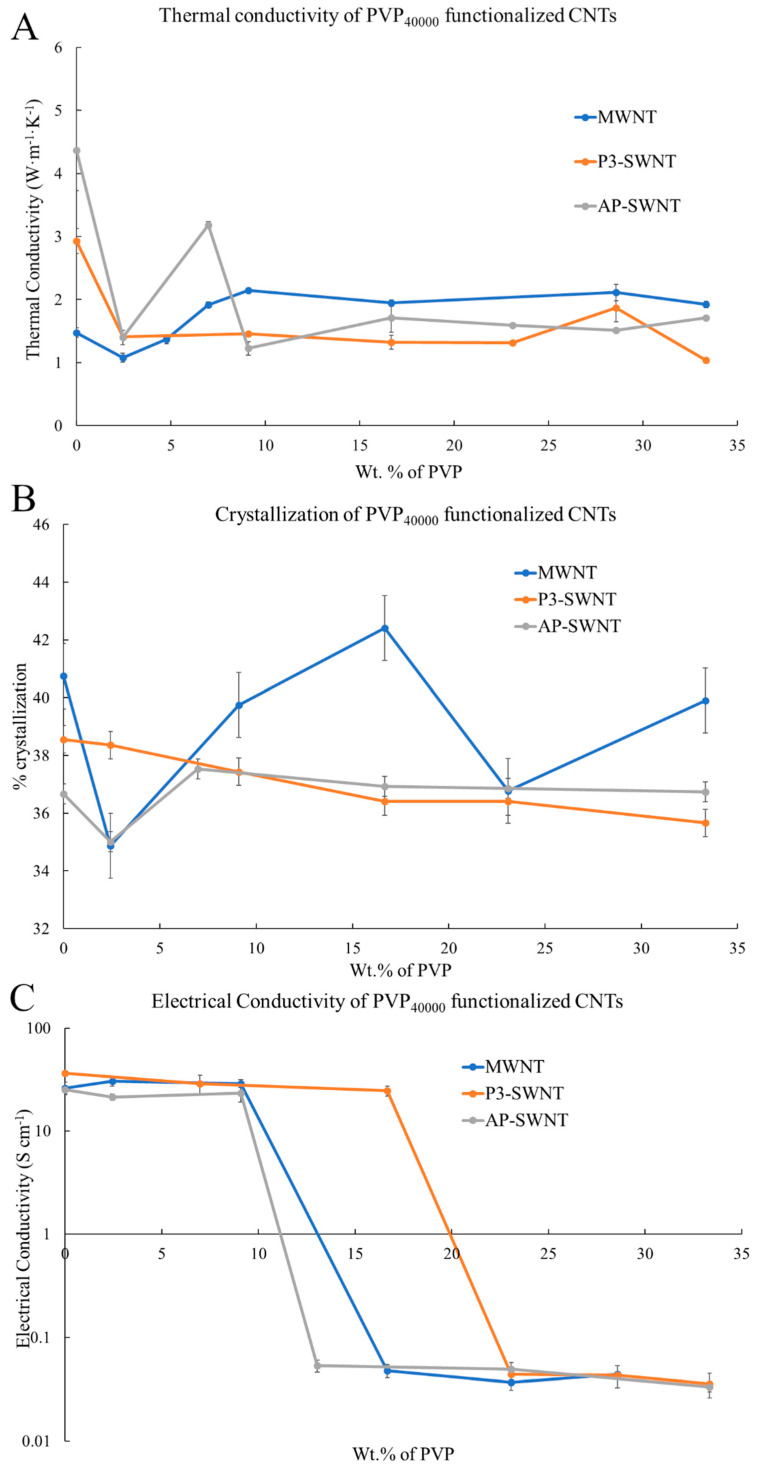
(**A**) Thermal conductivity of three different types of PVP_40000_ functionalized CNT/PVDF composites; (**B**) Percent Crystallization; and (**C**) Electrical conductivity of PVP_40000_ functionalized CNT/PVDF composites at different PVP concentrations.

An effective enhancement in thermal conductivity of about 45.23% was observed in the PVP_40000_ functionalized MWNT based composite compared to the unmodified nanotube composite. An increase in thermal conductivity from a value of about 1.477 W·m^−1^·K^−1^ to a value of about 2.145 W·m^−1^·K^−1^ was observed at a PVP concentration of 9.09 wt. %. However, PVP_40000_ functionalization could not initiate a similar effect in the AP-SWNT and P3-SWNT based composites. The result observed for AP-SWNT in Figure 3A confirms that the PVP_40000_ polymer chain was not compatible with the high aspect ratio of AP-SWNTs to achieve a homogeneous dispersion of nanotubes in the polymer matrix that could lead to the formation of a conductive nanotube network. Although, for AP-SWNT, it was observed from Figure 3A,B that a hike in thermal conductivity and dispersion was observed with PVP_40000_ functionalization at a concentration of 6.98 wt. %, it did not initiate dispersion in the overall polymer composite. This could be attributed to the fact that different lengths of polymer chain lead to different amount of surface coverage. The key is to achieve enough surface coverage of nanotubes through polymer wrapping that enhances nanotube dispersion in the matrix without disrupting the nanotube network formation. Similarly, the PVP_40000_ functionalization does not tend to initiate a homogeneous dispersion with P3-SWNT based composites because the surface area of the nanotubes could not be covered by PVP_40000_ in the low concentration range, but a small hike in thermal conductivity is observed at a concentration of 28.57 wt. %, as observed with PVP_10000_ functionalization of P3-SWNTs in Figure 1A.

The crystallization results observed in Figure 3B also confirmed that PVP_40000_ functionalization only produced an effective dispersion of nanotubes with MWNT based composite at a concentration range lower than 33 wt. %. However, PVP_40000_ functionalization had a completely different effect on the P3-SWNT and AP-SWNT based composites in this concentration range. Although an increase in crystallization behavior of about 2.37% was observed in AP-SWNT based composites with PVP_40000_ functionalization confirming an enhancement in the degree of nanotube dispersion, the interaction between the polymer and nanotubes is not sufficient to initiate an enhancement in conductivity of the overall composite. However, P3-SWNT does not tend to exhibit any degree of enhancement in nanotube dispersion with PVP_40000_ functionalization, leading to a decrease in the crystallinity and conductivity of the composite.

The electrical conductivity result correlated with the result observed in Figure 3A,B. MWNT based composites exhibited an increase in electrical conductivity from a value of about 26.1 S·cm^−1^ to about 30.6 S·cm^−1^ with PVP_40000_ functionalization at low concentration, followed by a decline at higher PVP concentrations (Figure 3C). However, a decrease in electrical conductivity compared to the unmodified nanotube composite was observed with PVP_40000_ functionalization in P3-SWNT and AP-SWNT based composites at low concentrations. However, a threshold point was reached with AP-SWNT quickly at low concentration, but P3-SWNT reached a threshold point at a comparatively higher concentration. Although P3-SWNT exhibited a lower conductivity compared to the unmodified nanotube composite, a stable conductivity was maintained until a threshold point is reached. This could be explained by the fact that PVP_40000_ functionalization has not created an impact with P3-SWNT dispersion in the polymer matrix at concentrations lower than 33 wt. %. The concentration of PVP_40000_ with its polymer chain length is not sufficient to functionalize an adequate number of P3 single-walled nanotubes in order to create a stable dispersion in the overall composite, but increasing the length of the polymer chain or increasing the concentration of the polymer for the same amount of P3 single-walled nanotubes would likely have a positive effect on the degree of nanotube dispersion in the polymer matrix.

**Figure 4:**
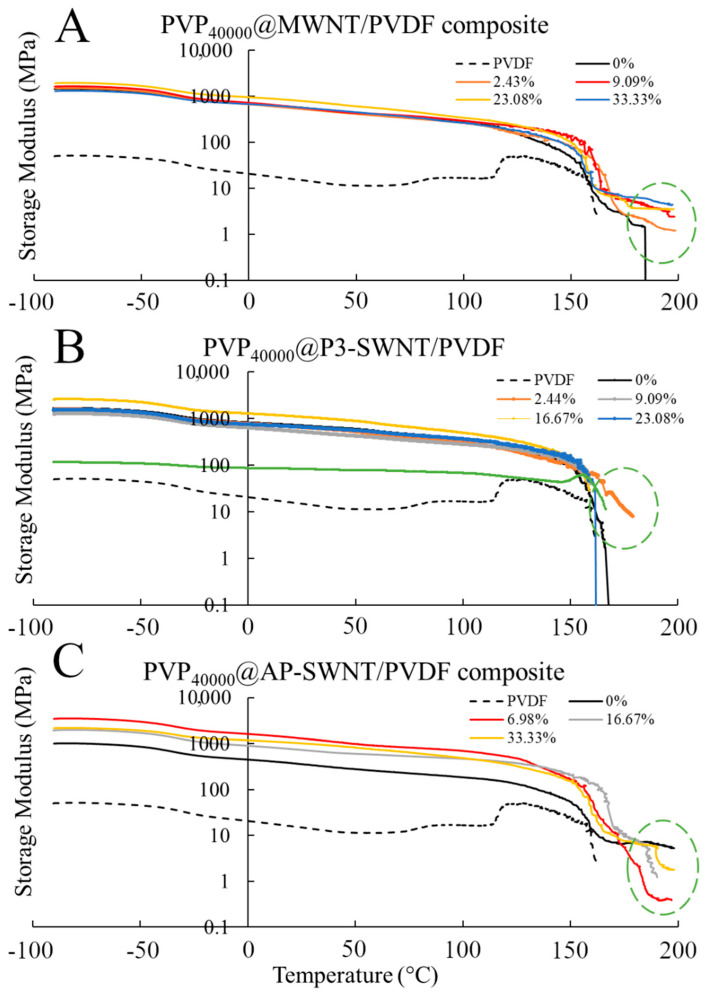
Storage Modulus of PVP_40000_ functionalized MWNT (**A**); P3-SWNT (**B**); and AP-SWNT (**C**) in PVDF composite.

The above-mentioned thermal, electrical, and crystallization results are reflected in the mechanical strength of the respective composites shown in Figure 4, such as the formation of an effective nanotube network observed in PVP_40000_ functionalized MWNT composites. Thermal conductivity enhancement in MWNT based composite is observed at a PVP concentration of 9.09 wt. % and higher, and the corresponding mechanical characterization results observed in Figure 4A confirmed that the storage modulus of these composites are higher than the unmodified nanotube composites and also exhibit a strong mechanical strength at higher temperatures (highlighted in green dashed circle). This confirmed an effective dispersion of nanotubes and the presence of a strong nanotube network, but such formations were not observed in P3-SWNT and AP-SWNT based composites. A low mechanical strength compared to unmodified nanotube composites was observed with AP-SWNT based composites, whereas P3-SWNT exhibited no effective mechanical strength above the yielding point of the unmodified nanotube composite. This confirmed the hypothesis that a PVP_40000_ concentration lower than 33 wt. % was not enough to achieve a dispersion sufficient to create a nanotube network that holds the composite together.

### 3.3. PVP_55000_ Functionalized CNT in PVDF Composite

An effective dispersion of nanotubes was observed through PVP_55000_ functionalization in MWNT based composites with a 59.85% increase in thermal conductivity at a concentration of 23.08%, and the P3-SWNT based composites exhibited a 45.65% increase in thermal conductivity in the same concentration range. This result confirmed the compatibility of the longer chain length with nanotubes of both higher and lower diameters. However, the result observed with AP-SWNT based composites exhibited only a 6% increase in thermal conductivity with PVP_55000_ functionalization. The longer nanotube length and higher aspect ratio of AP-SWNTs compared to MWNTs and P3-SWNTs could have led to a different amount of surface coverage and a different degree of nanotube dispersion in the polymer matrix with PVP_55000_ functionalization. This could have allowed a small enhancement in the thermal conductivity of the composite with PVP_55000_, but not one that was as effective as that observed with PVP_10000_ in Figure 1A.

**Figure 5:**
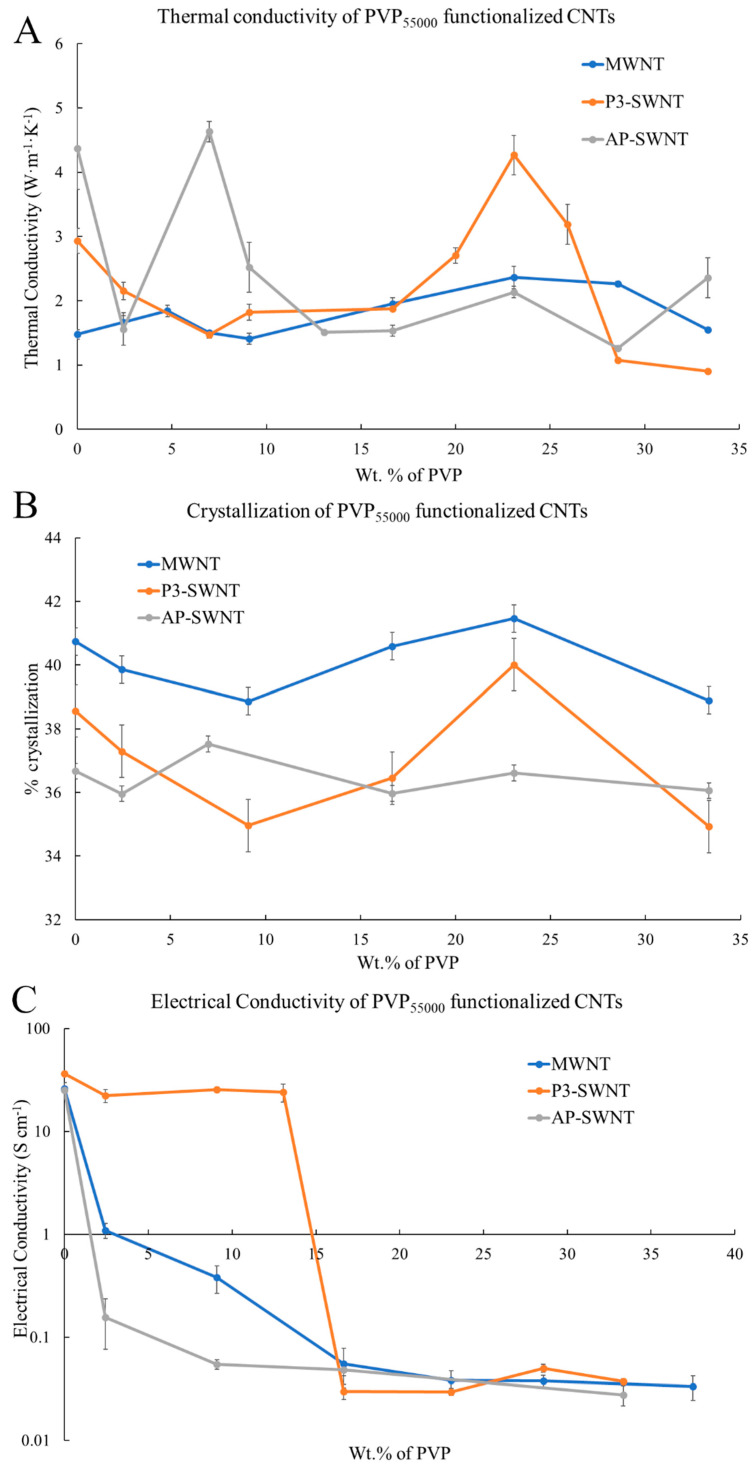
(**A**) Thermal conductivity of three different types of PVP_55000_ functionalized CNT/PVDF composites; (**B**) Percent Crystallization; and (**C**) Electrical conductivity of PVP_55000_ functionalized CNT/PVDF composites at different PVP concentrations.

The crystallization results correlated with the thermal conductivity observation in Figure 5A. The MWNT and P3-SWNT based composite exhibited an increase in the degree of crystallization of around 23.08 wt. % (Figure 5B), confirming a better interaction of the nanotubes and polymer matrix as a result of enhanced nanotube dispersion. AP-SWNT also exhibited an increase in the degree of crystallization at comparatively lower concentrations, as observed in the thermal conductivity graph.

The electrical conductivity result observed with PVP_55000_ functionalization of CNTs was different from the thermal conductivity and crystallization results. A decrease in electrical conductivity with increasing concentration of PVP_55000_ was observed in MWNT, owing to the fact that longer polymer chain leads to higher surface coverage of nanotubes, thus isolating neighboring nanotubes and compromising effective electron transport. It is to be noted that phonons have a tendency to travel along the sides of the nanotubes and through the polymer in order to reach the neighboring nanotubes, provided the layer of polymer is reasonably thin, but the layer of polymer would act as a hindrance for effective electron transport. This is confirmed in Figure 5C, with a decrease in electrical conductivity in PVP_55000_ functionalized MWNT composites along the same concentration range that exhibited a thermal conductivity enhancement. However, the PVP_55000_ functionalization in AP-SWNT led to an even higher surface coverage compared to MWNT based composites and, as a result, a very low thermal conductivity enhancement, as observed in Figure 5A, and further lower electron conductivity than MWNT based composites. Although good electron conductivity was observed with PVP_55000_ functionalization in P3-SWNT based composites at low concentrations, a decrease in conductivity was observed at higher concentrations, particularly in the concentration range that exhibits a thermal conductivity enhancement. This is due to the fact that the higher amounts of polymer present in the composite restricts the movement of electrons through the composite, leading to a poor conductivity, but polymer wrapping of nanotubes is imperative to achieve a stable dispersion. The key to achieving this without compromising the formation of an effective network is through achieving an effective dispersion at low PVP concentrations, leading to lower wrapping polymer presence and high connectivity between nanotubes.

**Figure 6:**
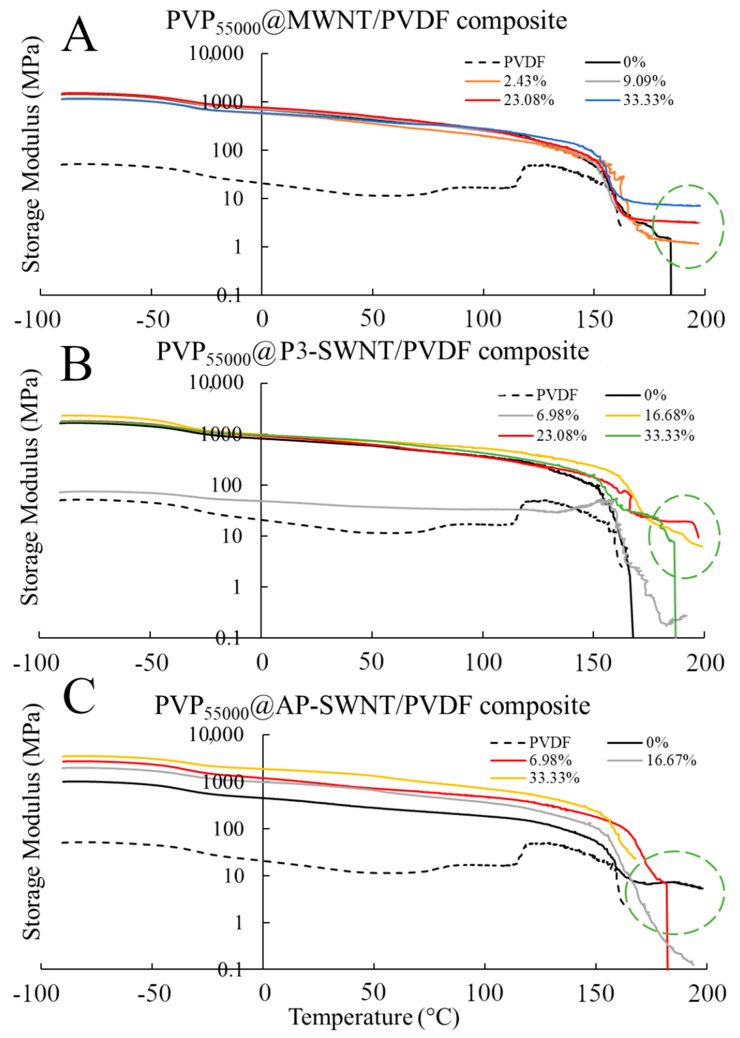
Storage Modulus of PVP_55000_ functionalized MWNT (**A**); P3-SWNT (**B**); and AP-SWNT (**C**) in PVDF composite.

The highest thermal conductivity value was observed with MWNT and P3-SWNT based composites functionalized with PVP_55000_ at a concentration of 23.08 wt. %, and these composites tend to exhibit a strong mechanical strength at high temperatures, as observed in Figure 6. Although the storage modulus of these composites was observed to be similar to an unmodified nanotube composite at low temperature range, they tend to maintain a stable network even beyond the yielding point of an unmodified nanotube composite, allowing the composites to withstand comparatively higher temperatures. This phenomenon was observed in PVP_55000_ functionalized MWNT composites at all concentrations higher than 9.09 wt. %, suggesting a higher degree of nanotube dispersion compared to the unmodified nanotube composite. However, a slightly different result was observed in P3-SWNT based composites. A higher degree of dispersion was observed only in composites prepared at a concentration of 23.08 wt. %, as shown in Figure 5A, and this composite shows a higher mechanical strength and withstands high temperatures. However, other PVP_55000_ functionalized composites exhibited a comparatively lower degree of dispersion, leading to either lower mechanical strength or lower yielding point.

PVP_55000_ functionalization promoted some degree of network formation in MWNT and P3-SWNT based composites that allowed a higher yield temperature compared to unmodified nanotube composites. However, such network formations were not observed with PVP_55000_ functionalization in AP-SWNT based composites, leading to a comparatively lower yielding temperature. The relation between chain length of the functionalization polymer and aspect ratio of AP-SWNT plays a key role in the outcome. A possible higher surface coverage of nanotubes with a longer chain length could have led to a disruption in the nanotube network, compromising nanotube connectivity and eventually resulting in a poor mechanical strength.

### 3.4. PVP_360000_ Functionalized P3-SWNT/AP-SWNT in PVDF Composite

**Figure 7:**
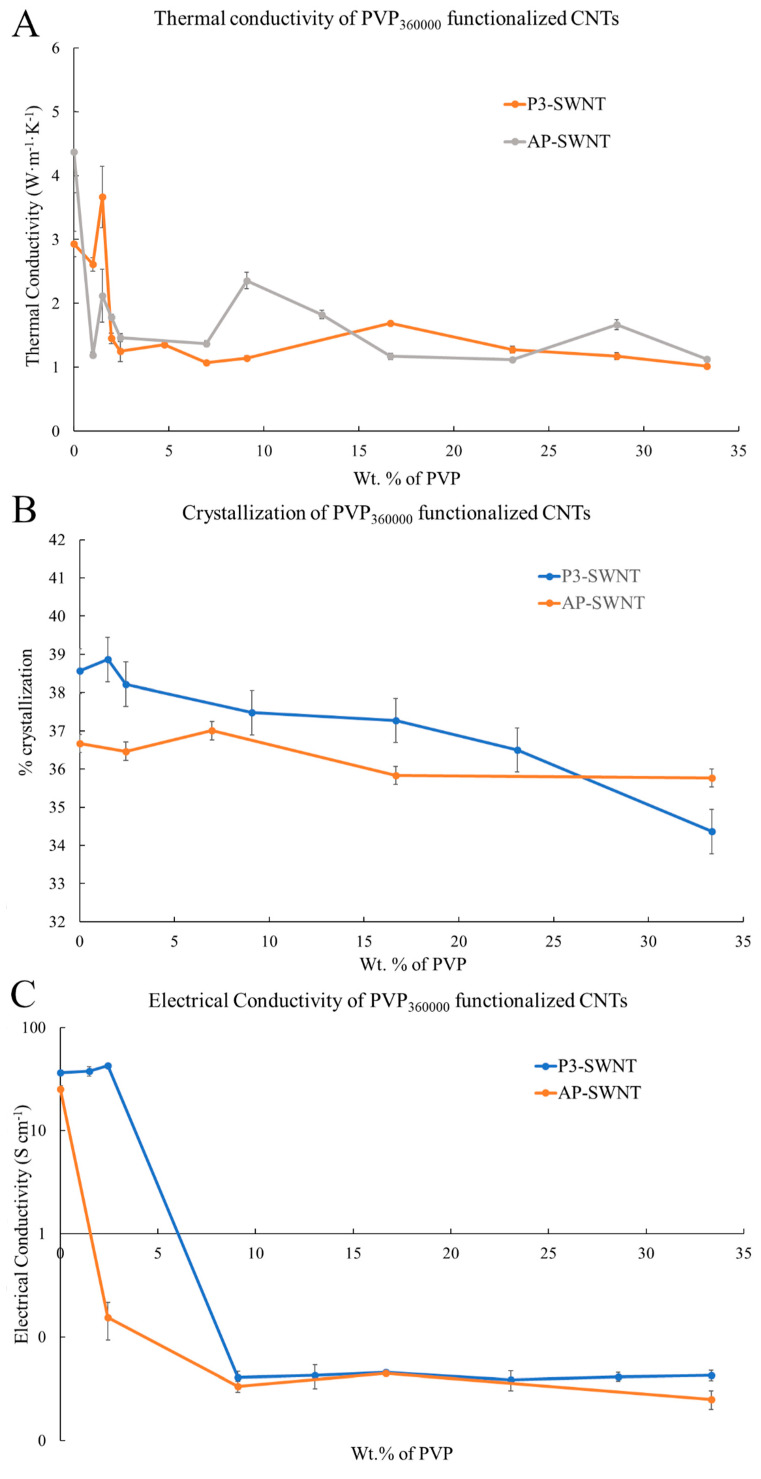
(**A**) Thermal conductivity of two different types of PVP_360000_ functionalized CNT/PVDF composites; (**B**) Percent Crystallization; and (**C**) Electrical conductivity of PVP_360000_ functionalized CNT/PVDF composites at different PVP concentrations.

The high molecular weight polymer showed an effective dispersion with P3-SWNT based composites. A 25.16% increase in thermal conductivity was observed at a very low concentration range of about 1.48 wt. % (Figure 7A). For polymer functionalization of nanotubes, the polymer needs wraps around the surface of the nanotube, forming a supra molecular complex. This can enhance the dispersibility of nanotubes in organic solvents, and this could be achieved by PVP_360000_ at a low concentration to create a positive impact towards the overall thermal conductivity of the composite. However, PVP_360000_ functionalization did create a similar impact over dispersion of a high aspect ratio AP-SWNT in the polymer matrix. The could be due to the fact that the number of individual nanotubes present in a certain amount (weight) of high aspect ratio AP-SWNTs would be substantially lower compared to P3-SWNTs of similar diameter but lower length, and eventually the number of individual nanotubes that needs to be functionalized in order to achieve an effective dispersion in the overall composite is also significantly altered (reduced). This would mean the longer polymer chain of PVP_360000_ might not be compatible in achieving a stable nanotube network in the same concentration range for AP-SWNTs, as observed in the thermal conductivity result with shorter polymer chain length (PVP_10000_) in Figure 1A. The longer polymer chain in this concentration range was adequate to functionalize the suspended AP-SWNTs, leading to excess functionalization of nanotubes, thus isolating the nanotubes from other neighboring nanotubes in the composite, making the phonon transport more difficult, resulting in low thermal conductivity, as observed in Figure 7A. However, the possibility of achieving an effective dispersion of AP-SWNTs with PVP_360000_ functionalization is high at an even lower PVP concentration, but this was not explored.

The thermal conductivity result is reflected in the crystallization behavior (Figure 7B) and the electrical conductivity (Figure 7C) of the PVP_360000_ functionalized nanotube composites. An improvement in the degree of crystallization was observed with P3-SWNT at low concentrations, confirming an effective dispersion of nanotubes, leading to a structured CNT network formation. Although a similar observation was witnessed with the crystallization behavior of AP-SWNT based composites, the dispersion of nanotubes did initiate a network structure formation, either due to excess surface coverage of a single nanotube or excess functionalization polymer in the composite. This might have led to the isolation of nanotubes from their neighbors, thus disrupting the connectivity between the nanotubes, leading to the absence of a conductive path. This was also reflected in the electrical conductivity result observed in Figure 7C. A decrease in electrical conductivity was observed with PVP_360000_ functionalization in AP-SWNT at low concentrations and kept decreasing with increasing PVP concentration until a percolation threshold was reached at around 9.09 wt. %., whereas a 16.44% increase in electrical conductivity was observed with PVP_360000_ functionalization in P3-SWNT composites at low concentrations that also exhibited a thermal conductivity enhancement.

**Figure 8:**
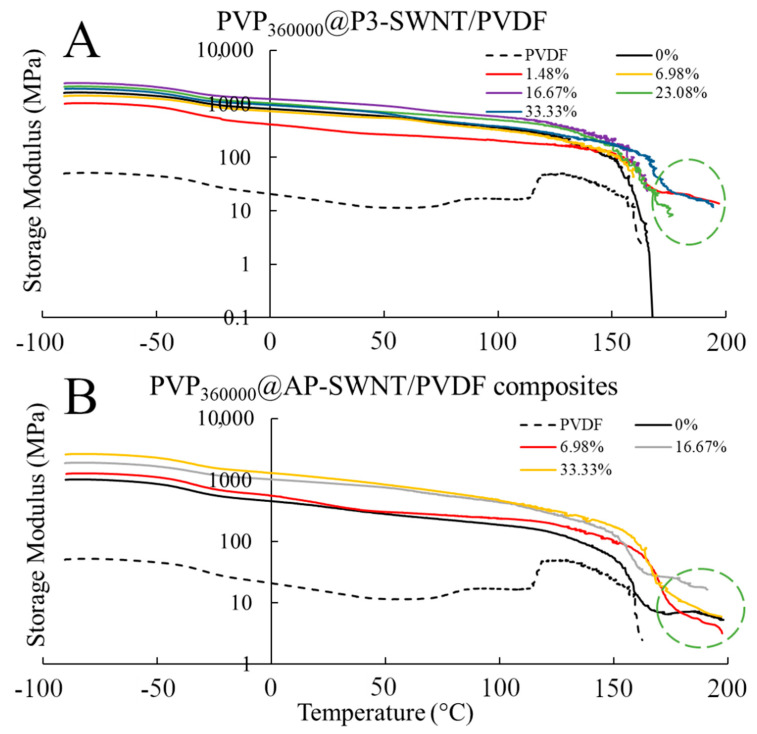
Storage Modulus of PVP_360000_ functionalized P3-SWNT (**A**) and AP-SWNT (**B**) in PVDF composite.

Mechanical characterization of the composite confirms the conductivity results observed in Figure 7A,C. For P3-SWNT, the composites prepared at a concentration of 1.48 wt. % showed a strong mechanical strength compared to the unmodified nanotube composite, as observed in Figure 8A, with an yielding temperatures as high as 197 °C, confirming the presence of some sort of nanotube network that holds the composite together. Other P3-SWNT composites of higher PVP concentration also exhibit a similar mechanical strength and a stable nanotube network, but they tend to sustain stability at a comparatively lower temperature. The high aspect ratio of AP-SWNT meant that their composites exhibited a strong mechanical strength at high temperatures and were able to sustain a stable network at temperatures as high as 198 °C. While PVP_360000_ functionalized AP-SWNT composites tend to yield at similar temperatures, as shown in Figure 8B, the stability of the nanotube composites compared to unmodified AP-SWNT composites was not observed to be improved through PVP_360000_ functionalization.

The results showed that nanotubes of different structures achieve different degrees of dispersion in the polymer matrix based on the molecular weight of the PVP functionalization polymer. MWNT based composites achieved dispersion through PVP functionalization at low concentrations for lower molecular weight and at comparatively higher concentrations with increasing molecular weight. This can be explained by the fact that the short polymer chains were capable of functionalizing a considerable number of nanotubes in the composites without compromising the connectivity at low PVP concentrations. However, only a limited number of nanotubes are wrapped at low concentration with longer polymer chains, and an increasing concentration of PVP is required to functionalize a significant fraction of the nanotubes to create an impact in the overall composite. The larger diameter nanotubes could have allowed longer polymer chain PVP to engage in a double or triple helical wrapping, requiring a higher concentration of PVP in order to cover all the individual nanotubes to create a supramolecular complex, which a small polymer chain could achieve at low concentration.

A similar phenomenon was observed by Karpushkin et al. on CNT dispersions in an aqueous medium assisted by wrapping with poly-N-vinylpyrrolidone. It was revealed that an efficient dispersion of CNT was not achieved at too low a PVP loading, because there was not enough polymer to wrap the nanotubes effectively and, while at a concentration of PVP that was too high, the dispersion was not the most efficient [43]. Huang et al. conducted a study on the mechanical properties of PVP wrapped MWNTs in a polyvinyl alcohol (PVA) matrix and reported that PVP facilitated uniform distribution of nanotubes in PVA hydrogels while building up a strong interface between nanotube and matrix. A 133% increase in tensile strength and a 43% increase in elongation-at-break has been observed with PVP functionalized MWNT/PVA composite of 1.0 wt. % MWNT, but it declines quickly with changing MWNT content. Additionally, the well dispersed MWNTs sustain excellent wetting in the PVA hydrogel matrix, leading to the formation of a denser network structure with smaller pores, resulting in enhanced thermal conductivity and crystallization of PVA [8]. A study on nanocomposite films of PVDF filled with PVP coated MWNT conducted by El Achaby et al. reported that enhanced thermal stability is observed in nanocomposite samples containing 0.1 wt. % of PVP-coated MWNTs, and the stabilization effect is witnessed for nanocomposites containing up to 0.5 wt. % of PVP-coated MWNT, but a dramatic decrease is observed at 1 wt. % and 2 wt. % containing samples, confirming that only certain combinations of initial concentration of CNTs and PVP in the mixture leads to enhanced thermal properties [37].

A contradicting result compared to MWNT results was observed with P3-SWNT based composites. A supramolecular complex with P3-SWNT at low PVP concentrations was achieved with a PVP of longer polymer chain, leading to higher degree of dispersibility followed by lower polymer chain length, exhibiting a similar effect at a comparatively high concentration range. The smaller diameter of P3-SWNTs compared to MWNTs could have altered the wrapping behavior, meaning that to achieve surface coverage of P3-SWNTs for effective functionalization with a longer polymer chain PVP required low concentrations to establish a higher degree of dispersion. Shorter polymer chains could only initiate a similar effect at higher concentrations.

High aspect ratio nanotubes without surface functionalization were effective in the formation of a nanotube network in the polymer matrix, thus exhibiting improved thermal, electrical, and mechanical properties, as observed with the AP-SWNT based composite. Although AP-SWNTs and P3-SWNTs have a similar diameter, the higher length of AP-SWNTs lead to an entirely different result. A higher degree of dispersion with formation of an effective nanotube network is achieved only through nanotube functionalization with a PVP of lower molecular weight. While a PVP of higher molecular weight tended to initiate a stable dispersion of nanotubes in the composite, they failed to achieve an effective network formation, leading to poor thermal, electrical, and mechanical properties. Surface coverage of nanotubes plays a key role because, despite the fact that the higher polymer chain length can aid in a higher dispersion of nanotubes, excess surface coverage tends to disrupt the network formation. It is better to achieve a higher degree of nanotube dispersion at a low PVP concentration range by understanding the relation between the nanotube structure and the molecular weight of the polymer. This allows the nanotubes to disperse effectively in the polymer matrix and yet form a network structure that enhances the thermal, electrical, and mechanical properties of the composite. This is observed in Table 1 with MWNT and AP-SWNT based composites functionalized with PVP_10000_ at a concentration of 2.44 wt. % and 6.98 wt. %, and P3-SWNT based composites functionalized with PVP_360000_ at a concentration of 1.48 wt. %.

## 4. Conclusions

An effective dispersion of MWNTs in the polymer matrix is achieved through PVP functionalization of low molecular weight (PVP_10000_) at a low PVP concentration range, leading to enhanced thermal, electrical, and mechanical properties compared to PVP of higher molecular weight, and a similar outcome is observed with AP-SWNT based composites at low PVP_10000_ concentrations. However, P3-SWNT based composites exhibited a different outcome such that an effective dispersion at low PVP concentration can be achieved only with a high PVP molecular weight (PVP_360000_). This difference in the outcome is attributed to the difference in the structure of nanotubes and its relation with the molecular weight (polymer chain length) of the functionalization polymer. Knowledge regarding the relationship between the structure of nanotubes and the molecular weight of the wrapping polymer is crucial in achieving non-covalent functionalization and an effective dispersion of nanotubes in a polymer matrix. The interplay between these two parameters is complex, but it is clear that the right combination of materials at the appropriate concentrations can lead to considerable enhancement of the composite properties.

## Figures and Tables

**Table 1:** Degree of enhancement/diminution in the thermal, electrical, and crystallization properties of the CNT-PVDF composite due to PVP functionalization of CNTs. The value provided for the thermal conductivity is the greatest enhancement observed. The number in brackets is the PVP concentration in weight percent at which this enhancement occurs. The electricity conductivity and % crystallization is provided for the same concentration for each system.

Carbon Nanotube Structure			MWNT	P3-SWNT	AP-SWNT
Functionalization Polymer	PVP 10000	Thermal Conductivity	126% (2.44 wt. %)	1.4% (9.09 wt. %)	53% (6.98 wt. %)
		Electrical Conductivity	−0.3%	−25.2%	9.9% ^#^
		Percentage of Crystallization	5%	−4.1%	3.1%
	PVP 40000	Thermal Conductivity	45.2% (9.09 wt. %)	−36.2% (28.6 wt. %)	−27.1% (6.98 wt. %)
		Electrical Conductivity	10.7%	−99.8%	−7.5% ^#^
		Percentage of Crystallization	−2.4%	−5.6% ^#^	2.3%
	PVP 55000	Thermal Conductivity	59.8% (23.1 wt. %)	45.6% (23.1 wt. %)	6% (6.98 wt. %)
		Electrical Conductivity	−99.8%	−99.9%	−99.7% ^#^
		Percentage of Crystallization	1.7%	3.7%	2.3%
	PVP 360000	Thermal Conductivity	-	25.1% (1.48 wt. %)	−46% (9.09 wt. %)
		Electrical Conductivity	-	3.5%	−99.8
		Percentage of Crystallization	-	0.8%	0.9%

^#^ measured at the closest concentration.

## Data Availability

The data presented in this study are available on request from the corresponding author. The data are not publicly available due to further publications being prepared.
